# Hands-on experience can lead to systematic mistakes: A study on adults’ understanding of sinking objects

**DOI:** 10.1186/s41235-017-0061-8

**Published:** 2017-06-20

**Authors:** Ramón D. Castillo, Talia Waltzer, Heidi Kloos

**Affiliations:** 1 Centro de Investigación en Ciencias Cognitivas, Universidad de Talca, Avenida Lircay s/n, Talca, CP 3460000 VII Región Chile; 2 Department of Psychology, University of California, Santa Cruz, 1156 High Street, Santa Cruz, CA 95064 USA; 3 Center for Cognition, Action, and Perception, Department of Psychology, University of Cincinnati, 5130 Edwards One, Cincinnati, OH 45221-0376 USA

**Keywords:** Naïve performance, Buoyancy, Embodied cognition

## Abstract

In line with theories of embodied cognition, hands-on experience is typically assumed to support learning. In the current paper, we explored this issue within the science domain of sinking objects. Adults had to make a guess about which of two objects in a pair would sink faster. The crucial manipulation was whether participants were handed real-life objects (real-objects condition) or were shown static images of objects (static-images condition). Results of Experiment 1 revealed more systematic mistakes in the real-objects than the static-images condition. Experiment 2 investigated this result further, namely by having adults make predictions about sinking objects after an initial training. Again, we found that adults made more mistakes in the real-objects than the static-images condition. Experiment 3 showed that the negative effect of hands-on experiences did not influence later performance. Thus, the negative effects of hands-on experiences were short-lived. Even so, our results call into question an undifferentiated use of manipulatives to convey science concepts. Based on our findings, we suggest that a nuanced theory of embodied cognition is needed, especially as it applies to science learning.

## Significance statement

Providing students with manipulatives and hands-on experiences is a common strategy to aid science learning. However, while haptic explorations provide richly concrete, multi-modal information about the domain, they can also mask underlying science concepts. In the current paper, we seek to add to this conversation, focusing specifically on the domain of sinking objects. Adults were given the opportunity to hold and manipulate various transparent containers that differed in size and number of weights. Their task was to predict which of two containers would sink faster in water. Surprisingly, their performance was worse than that of adults who were presented with static images of containers. It appears that hands-on experiences solidified systematic mistakes about how an object’s heaviness relates to its sinking rate. While these effects disappeared when real-life objects were replaced with static images, our findings nevertheless caution against an indiscriminate use of hands-on manipulatives. A carefully calibrated setup might be needed instead, namely to highlight relevant science content over and above irrelevant features.

## Background

With a significant national interest in science, technology, engineering, and mathematics (STEM) education, research efforts are needed to understand how to better teach science concepts. One specific challenge is to help the learner see beyond the most obvious regularities to detect hidden, but scientifically valid, regularities. In the current paper we seek to add to the conversation, looking at whether haptic experiences can be helpful to support science learning. The specific domain of choice pertains to the physics that governs sinking objects. This domain has the advantage of encompassing everyday occurrences, while at the same time featuring some non-intuitive intricacies. Thus, it is an ideal domain to study the emergence of scientifically valid knowledge. In what follows, we first provide a brief overview of the literature on hands-on science learning. We then discuss research on learning about sinking objects.

### Science learning and hands-on experiences

Many studies lament the challenge of science learning, the claim being that students’ naïve responses to phenomena conflict with conceptions established by the scientific community (for reviews, see Murphy & Alexander, 2008; Pfundt & Duit, 1993). While the exact nature of students’ knowledge is still debated (cf., Smith, diSessa, & Roschelle, 1993), the challenges that come with science learning are indisputable. They are pervasively documented in all aspects of science, including physics (e.g., Edens & Potter, 2003; Lee & Law, 2001; Mazens & Lautrey, 2003; Park & Han, 2002; Pozo & Gomez Crespo, 2005), chemistry (e.g., Chiu, Chou, & Liu, 2002; Harrison & Treagust, 2001; Boo & Watson, 2001), biology (e.g., Mikkilä-Erdmann, 2001; Windschitl, 2001), and astronomy (e.g., Diakidoy & Kendeou, 2001; Vosniadou & Brewer, 1992). It is therefore urgent to develop more effective science pedagogy, compared to typical instruction (e.g., Ohlsson, 1999, 2000).

In recent years, the adoption of diverse and integrated approaches to STEM education have called for inclusion of a more “hands-on” approach to teaching. The idea is to go beyond conveying material in pictorial two-dimensional format and endorse a “head, heart, and hands” pedagogy. It emphasizes not only the need for students’ minds, but also for their emotions (hearts) and their haptic experiences (hands) to be part of learning (e.g., Carlson & Sullivan, 1999; Ferguson & Hegarty, 1995; Sipos, Battisti, & Grimm, 2008). In line with these suggestions, there is indeed evidence that hands-on activities help with learning (e.g., Kontra, Goldin-Meadow, & Beilock, 2012; Kontra, Lyons, Fischer, & Beilock, 2015). For example, hands-on experiences in middle-school students’ science classes predict better science performance on a standardized test of achievement (Stohr-Hunt, 1996).

The call for hands-on activities has been further fueled by theoretical and empirical advances in the area of embodied cognition (e.g., Chemero, 2011; Gibbs, 2005; Wilson, 2002; Wilson & Clark, 2009). Proponents of the embodied-cognition theory claim that higher-level cognition is influenced by our bodily experience (e.g., Barsalou, 2008; Louwerse, 2007; 2008; Smith, 2005). And there is extensive empirical evidence to support this claim (for reviews, see Gibbs, 2005; Iverson & Goldin-Meadow, 2005; Spivey, 2008). It has lent credence to the pedagogical procedure of allowing the learner to actively experience real-life objects (e.g., Bilgin, 2006; Case & Fraser, 1999; Kahle & Damnjanovic, 1994).

At the same time, despite the optimism about hands-on learning, an unconditional endorsement of hands-on experiences is not confirmed unequivocally. For example, a study comparing learning in a fluid-mechanics course through video versus hands-on implementation found that students who watched videos performed just as well on assessments, or even better than the students who had hands-on experience (Abdel-Salam, Kauffman, & Crossman, 2006). Indeed, there has long been a debate over the efficacy of active hands-on activities versus static schematics in teaching science (e.g., Ma & Nickerson, 2006; McNeil & Jarvin, 2007; McNeil, Uttal, Jarvin, & Sternberg, 2009). In a commentary on the usefulness of concrete materials for learning, Brown, McNeil, and Glenberg (2009) caution against making the general assumption that concrete experience always leads to better learning. Rather, hands-on experience might sometimes relate to better learning, while at other times it may be unrelated to learning (Kirschner, Sweller, & Clark, 2006).

### Understanding the physics of sinking objects

To better understand the role of hands-on experiences, we focused specifically on the physics domain of sinking objects. Inhelder and Piaget (1958) were among the first to look systematically at the development of people’s understanding of sinking and floating. They presented children with a series of everyday objects (e.g., utensils, tools, toys, materials) and asked them to decide whether they would sink or float in water. Since then, the range of tasks employed in this domain has expanded considerably. It includes making predictions about a single object (e.g., Kohn, 1993; Rappolt-Schlichtmann, Tenenbaum, Koepke, & Fischer, 2007; Skoumios, 2009; Unal, 2008), comparing pairs of objects (e.g., Castillo, Kloos, Richardson, & Waltzer, 2015; Kloos & Somerville, 2001; Penner & Klahr, 1996), and providing explicit explanations about predictions (e.g., Hsin & Wu, 2011; Meindertsma, 2014; Smith, Carey, & Wiser, 1985).

Overall, findings are typically taken to imply the presence of mistaken beliefs about sinking objects (e.g., Butts, Hofman, & Anderson, 1993; Chinn & Malhotra, 2002; Hardy, Jonen, Möller, & Stern, 2006; Kang, Scharmann, & Noh, 2004; Kloos & Somerville, 2001; Skoumios, 2009; Unal, 2008). For example, participants in Penner and Klahr’s (1996) study often picked the heavier object as the faster one - even after learning the inaccuracies of this strategy. This pattern of mistaken behavior was further corroborated by verbal responses about what determines the sinking behavior of objects: Predictions about sinking and floating appear to be focused on weight or size exclusively, rather than on mass distribution (e.g., Castillo & Kloos, 2013; Castillo et al., 2015; Smith et al., 1985; but see Kloos, Fisher, & Van Orden, 2010; Kohn, 1993; Rappolt-Schlichtmann et al., 2007). Thus, this domain is ideal to investigate science learning.

Many learning studies on the physics of sinking objects have incorporated hands-on experiences as part of their didactic choices (e.g., Kloos & Somerville, 2001). However, the efficacy of this choice is far from established. In fact, the separate effect of hands-on experiences is often confounded with effects of instruction or curriculum changes (e.g., Unal, 2008; Hardy et al., 2006; see also Klahr, Triona, & Williams, 2007). To our knowledge, the only sinking-objects study that looked at the relative effect of hands-on manipulations was with 5-year-olds and 6-year-olds (Butts et al., 1993). Those findings showed that hands-on manipulations did not lead to learning by themselves. Instead, only the combination of both instruction and hands-on manipulation showed improved learning. The goal of the current study was to expand on these findings and investigate the effects of hands-on experience versus viewing static images in adults.

### Overview of the current study

In the current study,^1^ adults had to predict which of two objects would sink faster in water. Objects were transparent containers that differed in their size and in the number of weights inside. They were combined into pairs in such a way that neither the number of weights nor the size of the container was predictive of relative sinking rate. Thus, in order to perform correctly, participants had to compare objects on the basis of a variable other than mass or volume. Our question was whether adults’ predictions are affected by the type of stimuli: Do real-life objects yield better or worse predictions than static images of the objects?

Experiment 1 investigated the role of real-life objects on naïve performance - prior to any training. Half of the participants were handed real-life objects that they could explore haptically (real-objects condition). The other participants were shown static images of the objects (static-images condition). In Experiment 2, we applied the same prediction task, but now looking at the performance of participants who had been given training beforehand about sinking objects. Finally, in Experiment 3, we looked at whether effects of hands-on experiences would persist when real objects are removed and replaced with static images.

## Experiment 1

Do hands-on experiences influence naïve performance? The goal of Experiment 1 was to examine whether individuals would perform differently when making predictions about real-life objects compared to static images. Adults participated in two conditions, the real-objects condition and the static-images condition. In each condition, they were asked to make predictions about which of two objects would sink faster in water. The setting mimics an educational context in which a science instructor brings along real-life objects and prompts the learner to make various predictions about them.

### Methods

#### Participants

For this and all subsequent experiments, participants were recruited from a Midwestern university. Following an Institutional Review Board (IRB)-approved procedure, they provided their consent for participation and received partial course credit in return. There were 28 participants in the real-objects condition (10 men, 18 women; mean age = 18.65 years; SD = 1.97), and there were 25 participants in the static-images condition (11 men, 14 women; mean age = 20.78 years; SD = 2.37).

#### Materials and apparatus

**Fig. 1:**
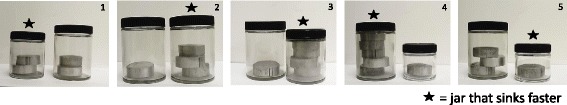
Examples of pairs of objects used for the predictions. Trials differ in whether the faster object in a pair was small (*1*), heavy (*2*), small and heavy (*3*), big and heavy (*4*), or small and light (*5*)

The objects were transparent glass containers that differed in size. Round aluminum discs could be placed inside the containers to obtain a desired density (see Appendix A for detailed dimensions). Depending on a container’s size and the number of weights inside, there were 12 unique objects. They were combined into pairs such that neither mass nor volume fully predicted the relative rate of sinking across all pairs. For example, in some pairs, the object that sank faster was the bigger and heavier container; and in other pairs, the object that sank faster was the smaller and lighter one. Figure 1 depicts several pairs to illustrate this point.

Real-life objects served as stimuli in the real-objects condition. For the static-images condition, we generated photographs of each unique pair of containers. A picture was 960 pixels wide and 720 pixels high. One picture showed two empty containers, each with a specific number of aluminum discs next to it. And the second picture showed the same two containers filled with the aluminum discs and closed with lids.

#### Procedure

Participants were tested individually in the laboratory, using DirectRT Precision Timing Software (2012 Version) to randomize the trials and record participants’ responses. Prior to the experiment proper, participants were introduced to the stimuli. They were first shown three empty containers of different sizes and several aluminum discs. They were then shown an image of two containers with discs inside them. They were told that the image represented a picture taken of the real objects in front of them. Next, participants were introduced to the task of predicting which of the two objects would sink faster when dropped in a tank of water. Participants’ prior knowledge about buoyancy was not assessed. No explanation was given about the underlying physics or how the participants should go about solving the task. The experiment started immediately.

There were 45 unique pairs of objects. Each possible pair was presented twice (with counter-balanced left-right position of objects). This yielded a total of 90 trials. The trials were presented in random order, with the caveat that a full set of 45 unique pairs was presented first, before any pair was repeated with its counterbalanced version.

**Fig. 2:**
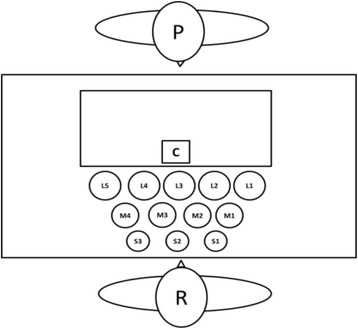
Setup for the real-objects condition. It features the 12 objects in front of the researcher (*R*) and an opaque box with a camera (*C*) in front of the participant (*P*)

In the real-objects condition, participants sat across from the researcher and in front of an opaque box that separated them (60 × 25 × 40 cm). The box served as a barrier behind which the researcher kept all 12 containers. Figure 2 provides a schematic overhead view of this arrangement. For each trial, objects were placed in the participant’s hands, and the participant had to choose the object they thought would sink faster. There was no time restriction for making a decision. After the participant made a choice, the experimenter recorded the choice on the computer and removed the containers from the participant’s hands. This ended the trial.

For the static-images condition, participants sat in front of the computer screen to view the images. Participants made their predictions using the keypad that had two marked choices (“left” and “right”). A trial started with the program presenting an image of two empty containers, each next to its respective stack of discs. After 1.5 seconds, the image was replaced with an image of the same two containers filled with the discs. Participants then had to decide which of the two containers would sink faster. There was no time restriction for making a decision. The trial ended when the participant marked a choice on the keypad.

### Results and discussion

**Fig. 3:**
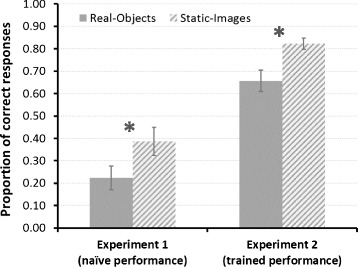
Proportion of correct responses on trials for which the faster object in a pair was small and light (see Panel 5 in Fig. 1). Results are separated by experiment and condition. *p < 0.05

We first looked at the data in terms of proportion of correct predictions. Across all possible trials, adults performed above chance (mean proportion *M*_real-objects_ = .77, *M*_static-images_ = .82). However, they made characteristic mistakes on trials in which the faster object was small and light (see Panel 5 in Fig. 1 for an example). There were 20 trials of this kind. Figure 3A provides the mean performance on these trials, separated by condition (see Appendix B for the data on all other trial types). Interestingly, performance in the real-objects condition (*M* = .22) was significantly lower than performance in the static-images condition (*M* = .39), independent-sample *t*(51) = 2.01, *p* < .05; *d*_Cohen_ = .55. Thus, hands-on experiences appear to have negatively impacted performance, leading participants to make more systematic mistakes when predicting the sinking rate of real objects. In fact, only performance in the real-objects condition, but not performance in the static-objects condition, was significantly below chance (assuming a chance probability of 0.5), *t*(25) = 5.27; *p* < .001, *d*_Cohen_ = 1.01.

In order to examine performance in more detail, we looked at individual performance over time. Specifically, we were interested in whether participants performed correctly, incorrectly, or randomly (throughout or eventually). Using a binomial-probability test, we identified nine patterns of responses (see Appendix C on how they were obtained). We then classified a person’s performance accordingly. Table 1 shows the number of participants per pattern of performance, separated by condition. As can be seen in the table, more participants performed incorrectly in the real-objects condition (75%) than in the static-images condition (56%). Vice versa, more participants performed correctly or randomly in the static-images condition (16%; 28%) than in the real-objects condition (7%; 18%). While these results did not reach statistical significance (one-tailed *χ* ^*2*^(1) = 2.13, *p* < .08), they nevertheless point in the same direction as the parametric results. Note that there was one participant in the real-objects condition who changed from performing consistently wrong to consistently right. However, there were also three participants in this condition who changed from performing consistently right to consistently wrong.

**Table 1:** Number of participants per pattern of performance in Experiment 1

Condition:	Correct (or eventually correct)			Incorrect (or eventually incorrect)			Random (or eventually random)		
	Random to correct	Incorrect to correct	Correct	Random to incorrect	Correct to incorrect	Incorrect	Random	Incorrect to random	Correct to random
Real objects	0	1	1	0	3	18	2	3	0
Static images	1	0	3	2	0	12	7	0	0

A binomial-probability test was carried out to classify a person’s performance on trials for which the faster object in a pair was small and light (see Appendix C for details)

Overall, we found that individuals made more mistakes when they predicted the relative sinking rate of real objects, compared to when making predictions with static images. There are two possible explanations for this finding: On the one hand, it is possible that hands-on experiences highlight misleading features. Perhaps the holding and hefting of real objects highlighted the feature of heaviness, over and above the more subtle feature of mass distribution. On the other hand, it is possible that real-life objects led to more stable learning - incorrect learning, but nevertheless learning. Maybe adults’ higher average in the static-images condition was not the result of some insight about sinking objects, but the result of simply guessing. Indeed, the average performance of participants in this condition is indistinguishable from chance. And while only 18% of participants in the real-objects condition performed randomly at any point during the experiment, a contrasting 40% of participants did so in the static-images condition (one-tailed *χ* ^*2*^(1) = 3.19, *p* < .04). Experiment 2 was carried out to disambiguate between the two possible explanations and clarify the effect of the real-objects manipulation.

## Experiment 2

Do hands-on experiences affect performance after training? The goal of Experiment 2 was to decide whether the presence of real-life objects highlights misleading features, or whether it has the benefit of stabilizing performance away from guessing. Towards this goal, we replicated Experiment 1 with one modification. The prediction task was now carried out after participants were given training about sinking objects. The training was picture-based and identical across conditions. It was immediately followed by the prediction task, with half of the participants being given real-life objects (real-objects condition) and the other participants being given static images (static-images condition). Our reasoning was that training would increase performance accuracy to above chance in both conditions. Any subsequent chance performance could then be attributed to a lack of learning. The setting in the real-objects condition is analogous to an educator providing a picture-based didactic intervention, after which students are presented with manipulatives to which they can apply the learned concepts.

### Methods

#### Participants

There were 28 participants in the real-objects condition (7 men, 21 women; mean age = 19.02 years, SD = 1.67) and 25 participants in the static-images condition (11 men, 14 women; mean age = 20.78 years, SD = 2.37).

#### Materials, apparatus, and procedure

There were two distinct phases in this experiment: a training session and a testing phase. Testing mimicked the method used in Experiment 1: Adults were presented either with real objects or with static images, and they were asked to decide which of two objects would sink faster in water. Prior to testing, participants took part in a training that was identical for both conditions. Specifically, the participants first made a prediction about which of two objects would sink faster. Then they received feedback about whether their prediction was correct or not. This type of training is known as predictive learning, supervised learning, or feedback learning (e.g., Garrison, Erdeniz, & Done, 2013; Van Hasselt, 2012). It mimics a pedagogy in which students are asked to generate an expectation and then test it explicitly. Materials for the training were static images of the sinking objects. Feedback was conveyed via an outcome image of one sinking object being ahead of the other in a water tank.

### Results and discussion

The feedback training was successful. To illustrate, we report average accuracy on participants’ predictions during the second half of the training. Across all trials, accuracy was near ceiling for both conditions (*M*_real-objects_ = .91; *M*_static-images_ = .92). Even when considering only trials for which the lighter and smaller object sank fastest, performance was above chance (*M*_real-objects_ = .82; *M*_static-images_ = .78, *p*s < .01). There was no difference between conditions during training, whether we considered the full set of trials, *F*(1,51) = 1.91; *p* > .17, or only the subset of trials for which the faster object was small and light, *t*(51) = 1.19, *p* > .24. An analysis of the 95% confidence interval confirmed the overlap (*CI*_real-objects_ = .82 ± .04; *CI*_static-images_ = 0.78 ± .05). The crucial question, then, was how participants performed after the training, when they were asked to make predictions either with real-life objects or with static images.

Past the training, adults performed close to ceiling across all trials (*M*_real-objects_ = .89, *M*_static-images_ = .91). The exception was their performance on trials in which the faster object in a pair was small and light (see Appendix B for the performance on all other trial types). Figure 3B depicts the mean accuracy on these trials, separated by condition. As we found in Experiment 1, performance was again lower in the real-objects condition (*M* = .66) than the static-images condition (*M* = .82), *t*(51) = 2.95, *p* < .001. Thus, even though participants demonstrated similarly high performance during training, the effect of real-life objects nevertheless emerged. Following the training, performance dropped significantly for participants in the real-objects condition, repeated-measure *t*(27) = 2.68, *p* < .02, while it increased slightly for participants in the static-images condition, repeated-measure *t*(24) = 2.31, *p* < .03.

Results from Experiment 2 reaffirm that hands-on experiences might highlight the heaviness of objects and thus lead to mistaken performance. In order to understand the seriousness of these effects, we next examined whether the negative influence of hands-on experiences would persist over time.

## Experiment 3

Does the mistake caused by hands-on experiences persist? When asked to predict the sinking rate of objects, we found that participants who were handed real-life objects made more mistakes than participants who viewed static images. We found this effect both in naïve performance and after training. When faced with these results, educators may wonder how much the use of manipulatives poses a concern for teaching effectively. To address this question, we examined whether mistaken performance lingered past an intermediate phase, when real-life objects were no longer present.

### Methods

#### Participants

Participants were the same as in Experiment 1: There were 28 participants in the real-objects condition (10 men, 18 women; mean age = 18.65 years, SD = 1.97) and there were 25 participants in the static-images condition (11 men, 14 women; mean age = 20.78 years, SD = 2.37).

#### Materials, apparatus, and procedure

After taking part in Experiment 1, participants were presented with the same feedback training that was used in Experiment 2: Images of pairs of objects were presented one by one, and participants received feedback on their predictions. Their predictions were then re-assessed with static images. Thus, there were three distinct phases of this experiment: a manipulation of hands-on versus static-image stimuli; a training session; and a test phase. If exposure to real-life objects has a long-term negative effect, even after training, we would expect to see a difference in performance between the real-objects condition and the static-images condition during the test phase.

### Results and discussion

We focused again only on trials in which the faster object is small and light (see Appendix B for the performance on all other trial types). Recall from Experiment 1 that naïve performance was below chance for both groups, and that participants in the real-objects condition performed worse than participants in the static-images condition. Following the training session, participants’ accuracy improved in both conditions, from .22 to .81 in the real-objects condition, *t*(27) = 10.34, *p* < .01, and from .39 to .82 in the static-images condition, *t*(24) = 6.71, *p* < .01. This suggests that the training was indeed helpful for overcoming the initial mistakes on trials in which the faster object is small and light. Importantly, the difference between the two conditions disappeared after the training session: During the test phase, performance in the real-objects condition was indistinguishable (*M* = .81) from performance in the static-images condition (*M* = .82), *t*(51) = 0.23, *p* > .82. Analyses of the 95% confidence intervals confirmed these results (*CI*_real-objects_ = .81 ± .05; *CI*_static-images_ = .82 ± .06). This shows that after an intermediate training session, participants were all able to reach an equally high level of accuracy, regardless of whether they were initially exposed to hands-on experiences.

Overall, we found that while hands-on experiences may initially lead to mistaken patterns of performance when making predictions about sinking objects, these mistakes could be overcome with training. We next turn to a general discussion of the findings from this research.

## General discussion

We set out to explore the influence of hands-on experience on learning the physics of buoyancy. Hands-on experience as a pedagogical tool has traction in the educational community. Its appeal is supported by the theoretical and empirical argument that cognition depends on the movement of our bodies (Abrahamson, 2014; Abrahamson, Gutiérrez, Lee, Reinholz, & Trninic, 2011; Kontra et al., 2012; Kontra et al., 2015). At the same time, some concerns have been voiced (e.g., Ma & Nickerson, 2006). This discrepancy warrants an explicit investigation into the relevance of hands-on experiences on learning. In the current study, we looked specifically at (1) whether hands-on experiences affect performance (Experiments 1 and 2) and (2) whether the effects persist after a delay (Experiment 3).

The results were clear: Despite using a setting that invites hands-on experiences (e.g., Flick, 1993; Haury & Rillero, 1994), we could not find support for claimed benefits. In fact, the opportunity for hands-on experiences, compared to viewing static images, led to more mistakes in both naïve performance and after training. It appears that hands-on experiences solidified systematic mistakes about how an object’s heaviness relates to its sinking rate. Thus, the beneficial effect of embodied experience was either absent or in the wrong direction. These findings undermine blanket claims about the advantages of hands-on, embodied learning. In what follows, we elaborate on this point.

### Why do embodied experiences hinder STEM learning?

One could argue that our manipulation in Experiment 2 had a confound: Participants who were given real-life objects had to switch from one type of stimulus to another (i.e., from static images used in the training session to real objects used in the test phase). By comparison, participants in the static-images condition might have had an advantage because they were already familiar with static images from the training. To rule out this possibility, it would be necessary to carry out the entire experiment with real-life objects. We had decided against this option because a lengthy feedback phase cannot be carried out feasibly with real-life objects. Note also that science-learning contexts typically employ images (e.g., in a text book) in addition to hands-on activities. This means, it is common for a learner to switch between manipulatives and images. Thus, a learning context carried out exclusively with real-life objects would have reduced ecological validity. In either case, differences in condition were already apparent in participants’ naïve performance during Experiment 1, before any switch in stimuli took place.

Although embodied experience failed to help STEM learning in our experiment, there are cases in which it does help (cf., Goldin-Meadow, Cook, & Mitchell, 2009; Goldin-Meadow & Wagner, 2005). It is possible that embodied experiences are useful if they provide better access to relevant information (cf., Kaminski, Sloutsky, & Heckler, 2008). In the context of sinking objects, the relevant information could be the distribution of mass or the degree of emptiness in the sinking container (Kloos & Van Orden, 2005). The empty space in our transparent containers was clearly visible. However, it would be difficult to feel empty space haptically. Thus, while mass distribution is available haptically in principle (e.g., Kloos & Amazeen, 2002), the hands-on experiences in our experiment were unlikely to afford participants with meaning, beyond what the viewing of static images could already provide.

Note that there is nothing inherently wrong with experiences that do not yield a measurable effect in learning. Some activities might simply serve the purpose of breaking up a dull learning event, like telling a joke during a lesson. A concern about such activities is only relevant if the experiences actually hinder learning. This is precisely what we found in our learning experiment: Adults exposed to real-life objects performed worse than adults exposed to static images. We consistently observed this effect both prior to and after a training session. Relevant information about mass and volume was available to both modalities: Participants could count the number of weights and compare the sizes of the containers in both conditions. Thus, to find a difference in performance as a function of condition is not trivial.

A possible explanation for the effect of condition is that real-life information added to task difficulty and thus yielded non-specific mistakes. This could follow from the idea that hands-on activities require dual representation, which can be more demanding than single representation (Ainsworth, 2006; Mayer & Moreno, 1998 Mayer & Moreno, 2003; McNeil & Jarvin, 2007). While plausible, this possibility is nevertheless unlikely. This is because the types of mistakes we found were systematic and specific. A difficult task would yield mistakes across all types of trials. Yet that is not what we found: Participants who were exposed to real-life objects did not demonstrate a general increase in mistakes. A possible increase in difficulty of the real-objects task therefore cannot explain the findings.

Another possibility is that the haptic experiences highlighted unnecessary aspects of the situation and masked relevant aspects. Such focus on irrelevant input might have interfered with participants’ efforts to analyze the pairs of objects carefully (cf., Kaminski et al., 2008; Son, Smith, & Goldstone, 2008). Without taking the time to compare the objects carefully, participants might have defaulted to the simplistic strategy of ignoring all but the most salient feature. However, this possibility also falls short on explaining the mistaken focus on heaviness. Differences in heaviness were likely to be less salient than differences in object size. In fact, the difference in mass between objects was very small and therefore relatively difficult - if not impossible - to be perceived haptically (cf., Weber, 1834/1978). And yet, the hands-on experience highlighted this feature of heaviness, not size.

It is possible that hands-on experiences, even without providing relevant information, could nevertheless change the landscape of salience across the entire perceptual system, beyond what is available haptically. For example, embodiment could affect perception that is outside of haptics and body movement. Such a spread of activation would imply that visual and embodied perception are interlinked: Behavior derived from embodied experiences might not be separable from behavior derived from other means of perception. This explanation aligns with approaches to the mind as a unified whole (e.g., Clark, 2013; Smith, 2005). Rather than think of movement as something independent or special, one could think of it as a component of learning and adaptive behavior, an aspect that could backfire when it highlights irrelevant features.

## Conclusion

In summary, the findings from this study underscore the nuanced nature of the interactions between embodied experiences and learning. We now know that hands-on experiences can elicit mistaken performance, such as in the domain of density and sinking objects. Indeed, hands-on activities may not always facilitate the best science learning outcomes. Thus, before deciding whether to incorporate hands-on activities in a curriculum, it is important to consider the added information that is provided by hands-on experiences. While our results do not lend themselves to specific recommendations for teachers, they nevertheless caution against an indiscriminant use of hands-on manipulatives. A carefully calibrated setup might be needed instead, namely to highlight relevant science content over and above irrelevant features.

## Figures and Tables

**Fig. 4:**
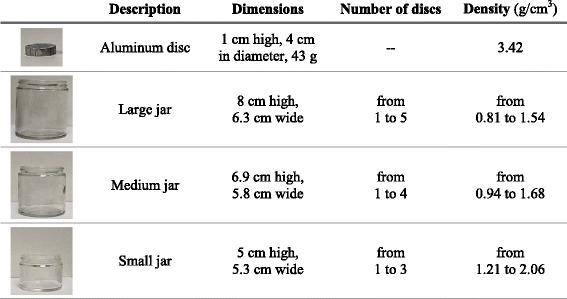
Specific dimensions of the materials used for the study

**Fig. 5:**
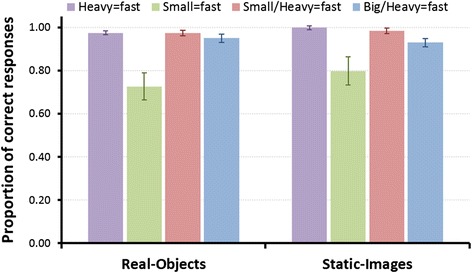
Average proportion of correct answers in Experiment 1, separated by condition and trial type (see Fig. 1 for an example of each trial type). Trials differ in whether the faster object in a pair was heavy (heavy = fast), small (small = fast), small and heavy (small/heavy = fast), or big and heavy (big/heavy = fast). A mixed-design condition-by-trial analysis of variance (ANOVA) revealed a main effect of trial type. Specifically, we found worse performance on small = fast trials (*M* = .76) and big/heavy = fast trials (*M* = .94) than on the other two trial types (*M*s > .98), *F*(3,153) = 18.88, *p* < .001, *η* ^2^ = 0.27, 1-*β* = .99.

**Fig. 6:**
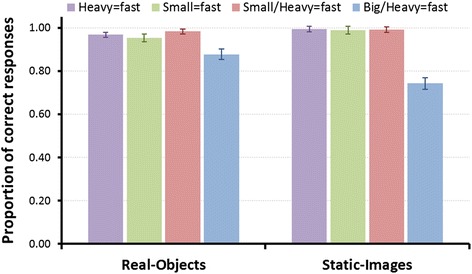
Average proportion of correct answers in Experiment 2, separated by condition and trial type (see Fig. 1 for an example of each trial type). Trials differ in whether the faster object in a pair was heavy (heavy = fast), small (small = fast), small and heavy (small/heavy = fast), or big and heavy (big/heavy = fast). A mixed-design condition-by-trial analysis of variance (ANOVA) revealed a main effect of trial type. Specifically, we found worse performance on big/heavy = fast trials (*M* = .88) than on all other trials (*M*s > .97), *F*(3,153) = 62.54, *p* < .001, *η* ^2^ = 0.55, 1-*β* = .99. There was also a significant condition-by-trial interaction, *F*(3,153) = 13.87, *p* < .001, *η* ^2^ = 0.21, 1-*β* = .99: In the static-images condition, only performance on big/heavy = fast trials (*M* = .74) was lower than all other trials (*M*s = .99). In contrast, in the real-objects condition, performance on both big/heavy = fast trials (*M* = .88) and small = fast trials (*M* = .95) was lower than the performance on all other trials (*M*s = .99). No other effects were significant, *F*(3, 153) < 0.86; *p* > .46.

**Fig. 7:**
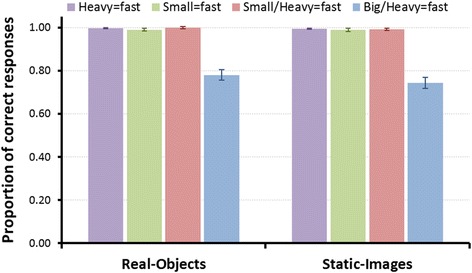
Average proportion of correct answers in Experiment 3, separated by condition and trial type (see Fig. 1 for an example of each trial type). Trials differ in whether the faster object in a pair was heavy (heavy = fast), small (small = fast), small and heavy (small/heavy = fast), or big and heavy (big/heavy = fast). A mixed-design condition-by-trial analysis of variance (ANOVA) revealed a main effect of trial type. Specifically, we found worse performance on big/heavy = fast trials (*M* = .76) than on all other trials (*M*s = .99), *F*(3,153) = 156.20, *p* < .0001, *η* ^2^ = 0.75, 1-*β* = .99. No other effects were significant.

**Fig. 8:**
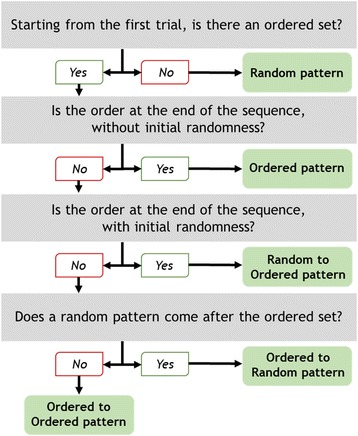
Decision tree to categorize patterns of performance in Experiment 1

## Appendix C

To determine what type of pattern best characterized a person’s responses, we used an incremental binomial-probability analysis. Non-random performance was defined as getting the lowest possible ratio of this set: 5/5, 6/6, 7/7, 7/8, 8/9, 9/10, 9/11, 10/12, 10/13, 11/14, 12/15, 12/16, 13/17, 13/18, 14/19, 15/20 (selected on the basis of the one-tailed binomial probability *p* < 0.05, assuming a chance probability of 0.5 per trial). Figure 8 shows the decision tree we followed to classify performances. Two authors of the paper classified the patterns independently of each other, yielding 100% agreement.

## Abbreviations

IRB: Institutional review board
STEM: Science, technology, engineering, and mathematics

## Footnotes

1 This study was part of a larger study (Castillo 2014) designed to investigate constraints on supervised and unsupervised learning. Adults participated in three phases in a single session (pre-test, training, post-test). Real-life objects were used during only one of the phases - if at all. Data from three groups of participants are reported: One group had real-life objects during the pre-test, one group had real-life objects during the post-test, and one group was presented with images of objects throughout. For ease of description, the presentation of these data is broken down into three experiments.
